# Supplementary material for: Frontal EEG Changes with the Recovery of Carotid Blood Flow in a Cardiac Arrest Swine Model
Source: Sensors (Basel). 2020 May 28;20(11):3052. doi: 10.3390/s20113052 (PMC7313692; doi:10.3390/s20113052)
Supplement: Supplementary file 1 [file sensors-20-03052-s001.zip › Supporting file S1_May25.docx]

File 1. Haemodynamic changes of individual animal throughout the experiments

| Test | Haemodynamic parameter (%) | Pre-VF | VF | BLS | | | | ACLS | | | | | | | | | | ROSC |
| --- | --- | --- | --- | --- | --- | --- | --- | --- | --- | --- | --- | --- | --- | --- | --- | --- | --- | --- |
|  |  |  |  | 1 | 2 | 3 | 4 | 1 | 2 | 3 | 4 | 5 | 6 | 7 | 8 | 9 | 10 |  |
| 1 | MAP | 100 | 36.2 | 66.3 | 69.9 | 71.6 | 69.7 | 71.9 | - | - | - | - | - | - | - | - | - | 80.2 |
|  | COPP | 100 | 15.2 | 51.4 | 54.9 | 56.5 | 54.7 | 57.0 | - | - | - | - | - | - | - | - | - | 43.6 |
|  | ETCO2 | 100 | 47.0 | 61.6 | 71.1 | 76.4 | 70.7 | 86.0 | - | - | - | - | - | - | - | - | - | 97.4 |
|  | CBF | 100 | 19.6 | 52.5 | 53.5 | 54.1 | 32.9 | 53.7 | - | - | - | - | - | - | - | - | - | - |
| 2 | MAP | 100 | 26.7 | 41.8 | 42.4 | 43.0 | 40.1 | 38.7 | 34.2 | 29.2 | 20.2 | 16.1 | 14.3 | 12.7 | 12.1 | 12.5 | 12.5 | X |
|  | COPP | 100 | 26.9 | 42.0 | 40.7 | 42.3 | 39.1 | 37.9 | 32.9 | 28.9 | 19.4 | 15.7 | 13.4 | 11.9 | 14.0 | 12.9 | 12.9 |  |
|  | ETCO2 | 100 | 46.6 | 49.4 | 47.2 | 48.9 | 45.7 | 49.0 | 46.0 | 35.6 | 24.2 | 13.2 | 10.5 | 13.3 | 12.8 | 11.4 | 11.1 |  |
|  | CBF | 100 | 2.9 | 37.1 | 27.0 | 23.1 | 18.0 | 15.1 | 10.0 | 8.1 | 2.5 | 1.1 | 0.7 | 0.1 | 0.0 | 0.1 | 0.1 |  |
| 3 | MAP | 100 | 13.8 | 43.0 | 35.7 | 26.6 | 25.9 | 39.0 | 33.0 | 29.0 | 28.4 | 26.7 | 23.4 | 45.3 | - | - | - | 62.9 |
|  | COPP | 100 | 11.4 | 30.6 | 49.9 | 67.7 | 26.6 | 36.3 | 31.8 | 28.6 | 28.1 | 26.8 | 24.3 | 40.9 | - | - | - | 54.6 |
|  | ETCO2 | 100 | 48.6 | 69.1 | 72.5 | 71.6 | 93.4 | 91.2 | 87.9 | 79.7 | 80.4 | 83.5 | 89.5 | 117.1 | - | - | - | 107.6 |
|  | CBF | 100 | 9.3 | 38.9 | 37.1 | 35.8 | 36.0 | 62.5 | 53.1 | 47.1 | 47.2 | 51.5 | 49.5 | 100.5 | - | - | - | 122.9 |
| 4 | MAP | 100 | 21.7 | 64.8 | 69.4 | 73.1 | 75.7 | 69.3 | 48.1 | - | - | - | - | - | - | - | - | 158.5 |
|  | COPP | 100 | 2.1 | 49.0 | 56.0 | 59.6 | 56.4 | 54.3 | 34.2 | - | - | - | - | - | - | - | - | 154.5 |
|  | ETCO2 | 100 | 53.8 | 72.7 | 86.8 | 94.4 | 100.3 | 86.2 | 98.6 | - | - | - | - | - | - | - | - | 147.7 |
|  | CBF | 100 | 17.8 | 59.8 | 77.0 | 109.2 | 113.4 | 71.5 | 59.5 | - | - | - | - | - | - | - | - | 111.0 |
| 5 | MAP | 100 | 25.5 | 34.4 | 35.3 | 34.7 | 35.4 | 32.3 | 28.7 | 26.1 | 23.1 | 18.0 | 14.1 | 10.2 | 7.7 | 7.3 | 7.3 | X |
|  | COPP | 100 | 8.5 | 20.9 | 20.7 | 19.1 | 18.4 | 17.2 | 13.9 | 14.9 | 10.6 | 5.7 | 3.1 | -1.3 | -1.6 | -1.2 | -1.2 |  |
|  | ETCO2 | 100 | 54.6 | 72.6 | 91.0 | 102.5 | 124.5 | 119.6 | 98.9 | 93.0 | 98.3 | 70.9 | 72.4 | 57.4 | 55.5 | 46.5 | 46.5 |  |
|  | CBF | 100 | 21.6 | 34.3 | 18.6 | 10.4 | 0.1 | 4.3 | 4.7 | 4.1 | 3.3 | 2.1 | 0.2 | -0.6 | -0.5 | -0.2 | -0.2 |  |
| 6 | MAP | 100 | 25.3 | 27.1 | 31.3 | 36.2 | 39.8 | - | - | - | - | - | - | - | - | - | - | 124.9 |
|  | COPP | 100 | 17.0 | 18.2 | 21.2 | 21.2 | 24.1 | - | - | - | - | - | - | - | - | - | - | 116.7 |
|  | ETCO2 | 100 | 44.9 | 90.5 | 98.8 | 106.4 | 115.0 | - | - | - | - | - | - | - | - | - | - | 116.1 |
|  | CBF | 100 | 27.4 | 30.2 | 37.4 | 39.9 | 37.6 | - | - | - | - | - | - | - | - | - | - | 109.8 |
| 7 | MAP | 100 | 29.9 | 46.3 | 59.6 | 57.9 | 60.0 | 69.1 | 69.3 | 64.9 | 67.4 | 64.4 | 62.3 | 66.0 | 64.9 | - | - | 71.3 |
|  | COPP | 100 | 25.6 | 49.6 | 62.1 | 56.6 | 61.1 | 66.1 | 69.4 | 66.4 | 67.6 | 68.0 | 65.6 | 66.4 | 66.8 | - | - | 81.1 |
|  | ETCO2 | 100 | 50.3 | 64.0 | 80.4 | 82.9 | 90.5 | 83.4 | 80.8 | 75.6 | 83.2 | 95.7 | 101.4 | 103.7 | 112.5 | - | - | 130.2 |
|  | CBF | 100 | 7.6 | 41.7 | 50.8 | 45.3 | 47.0 | 43.7 | 41.0 | 32.9 | 26.2 | 33.3 | 37.7 | 30.0 | 34.2 | - | - | 126.4 |
| 8 | MAP | 100 | 27.0 | 54.5 | 51.8 | 56.7 | 54.4 | 49.1 | 50.4 | 48.7 | 44.8 | 35.1 | 29.6 | 26.3 | 26.3 | 25.0 | 25.0 | X |
|  | COPP | 100 | 16.2 | 44.8 | 42.2 | 46.2 | 45.1 | 41.0 | 34.1 | 28.9 | 21.0 | 18.4 | 11.8 | 7.5 | 9.5 | 3.0 | 3.0 |  |
|  | ETCO2 | 100 | 52.8 | 58.3 | 66.5 | 95.2 | 66.5 | 66.0 | 79.1 | 90.2 | 84.9 | 90.4 | 72.3 | 83.6 | 100.5 | 114.9 | 114.9 |  |
|  | CBF | 100 | 6.5 | 28.3 | 27.8 | 11.3 | 29.2 | 25.2 | 21.5 | 15.3 | 17.7 | 17.1 | 16.3 | 17.0 | 18.1 | 20.1 | 20.1 |  |

Abbreviation: VF, ventricular fibrillation; BLS, basic life support; ACLS, advanced cardiovascular life support; ROSC, return of spontaneous circulation; MAP, mean arterial pressure; COPP, coronary perfusion pressure; ETCO2, end-tidal carbon dioxide; CBF, carotid blood flow.
