# Supplementary material for: Frontal EEG Changes with the Recovery of Carotid Blood Flow in a Cardiac Arrest Swine Model
Source: Sensors (Basel). 2020 May 28;20(11):3052. doi: 10.3390/s20113052 (PMC7313692; doi:10.3390/s20113052)
Supplement: Supplementary file 1 [file sensors-20-03052-s001.zip › Table1_May25.docx]

Table 1. EEG parameters considered in this study

| EEG parameters | Definition | Domain |
| --- | --- | --- |
| Magnitude | Maximal amplitude during the epoch (unit: µV) | Time |
| SynchFastSlow | log(B_0.5-47 Hz_ / B_40-47 Hz_) | Frequency |
| BetaR | log(P_30-47 Hz_ / P_11-20 Hz_) | Frequency |
| DeltaR | log(P_8-20 Hz_ / P_1-4 Hz_) | Frequency |
| AlphaPR | P_8-13 Hz_ / P_0.5-47 Hz_ | Frequency |
| BetaPR | P_13-30 Hz_ / P_0.5-47 Hz_ | Frequency |
| DeltaPR | P_0.5-4 Hz_ / P_0.5-47 Hz_ | Frequency |
| ThetaPR | P_4-8 Hz_ / P_0.5-47 Hz_ | Frequency |
| BG_Alpha+ | P_8-47 Hz_ / P_0.5-47 Hz_ | Frequency |
| Log energy entropy | $\sum_{i=1}^{n} {log(p\left( x_{i} \right))}^{2}$ | Entropy |
| Rényi entropy | $\frac{1}{1-\alpha}log(\sum_{i=1}^{n} {p\left( x_{i} \right)}^{\alpha}), (\alpha\geq0, \neq1)$ | Entropy |

Abbreviation: P_a-b Hz_, the sum of spectral power from a-b Hz; B_a-b Hz_, the sum of bispectral activity from a-b Hz; $\boldsymbol{p}\left( \boldsymbol{x}_{\boldsymbol{i}} \right)$, probability distribution function of signal $\boldsymbol{x}_{\boldsymbol{i}}$; $\boldsymbol{\alpha}$ of rényi entropy was 0.5;
