# Supplementary material for: Frontal EEG Changes with the Recovery of Carotid Blood Flow in a Cardiac Arrest Swine Model
Source: Sensors (Basel). 2020 May 28;20(11):3052. doi: 10.3390/s20113052 (PMC7313692; doi:10.3390/s20113052)
Supplement: Supplementary file 1 [file sensors-20-03052-s001.zip › Table2_May25.docx]

Table 2. Pearson correlation coefficients between EEG parameters and the recovery rates of CBF

| EEG parameters | Correlation  coefficient | P-value |
| --- | --- | --- |
| Magnitude | 0.778 | < 0.001 |
| SynchFastSlow | 0.210 | 0.228 |
| BetaR | -0.329 | 0.016 |
| DeltaR | 0.196 | 0.032 |
| AlphaPR | 0.189 | 0.048 |
| BetaPR | 0.323 | 0.001 |
| DeltaPR | 0.032 | 0.797 |
| ThetaPR | -0.354 | 0.004 |
| BG_Alpha+ | 0.262 | 0.006 |
| Log energy entropy | 0.781 | < 0.001 |
| Rényi entropy | 0.784 | < 0.001 |
