# Supplementary material for: Frontal EEG Changes with the Recovery of Carotid Blood Flow in a Cardiac Arrest Swine Model
Source: Sensors (Basel). 2020 May 28;20(11):3052. doi: 10.3390/s20113052 (PMC7313692; doi:10.3390/s20113052)
Supplement: Supplementary file 1 [file sensors-20-03052-s001.zip › Table3_May25.docx]

Table 3. Results of multiple comparisons between groups in three EEG parameters

|  | | Magnitude | Log energy entropy | Rényi entropy |
| --- | --- | --- | --- | --- |
| GroupⅠ/GroupⅡ | | Mean difference /  Standard deviation (P-value) | Mean difference /  Standard deviation (P-value) | Mean difference /  Standard deviation  (P-value) |
| 1 | 2 | -10.39 / 1.24  **(< 0.001)** | -1375.15 / 164.65  **(< 0.001)** | -2.69 / 0.319  **(< 0.001)** |
| 1 | 3 | -13.34 / 1.37  **(< 0.001)** | -1590.42 / 164.87  **(< 0.001)** | -3.13 / 0.321  **(< 0.001)** |
| 1 | 4 | -15.15 / 2.39  **(0.012)** | -1720.80 / 190.02  **(< 0.001)** | -3.38 / 0.434  **(< 0.001)** |
| 2 | 3 | -2.95 / 1.30  (0.169) | -215.27 / 87.24  (0.108) | -0.442 / 0.171  (0.084) |
| 2 | 4 | -4.75 / 2.35  (0.395) | -345.65 / 128.60  (0.180) | -0.695 / 0.338  (0.384) |
| 3 | 4 | -1.80 / 2.41  (0.958) | -130.39 / 128.87  (0.871) | -0.253 / 0.340  (0.957) |

Differences were obtained by GroupⅠ minus GroupⅡ.

Bold values denote statistical significance at the P < 0.05 level.
