# Supplementary material for: Frontal EEG Changes with the Recovery of Carotid Blood Flow in a Cardiac Arrest Swine Model
Source: Sensors (Basel). 2020 May 28;20(11):3052. doi: 10.3390/s20113052 (PMC7313692; doi:10.3390/s20113052)
Supplement: Supplementary file 1 [file sensors-20-03052-s001.zip › Table4_May25.docx]

Table 4. Results of the ROC curve analysis for EEG parameters

| EEG Parameter | AUC | Standard  error | True positive rate (Sensitivity) | False  positive rate  (1 - Specificity) | Cut-off value |
| --- | --- | --- | --- | --- | --- |
| Magnitude | 0.904 | 0.033 | 0.889 | 0.244 | 12.802 |
| Log energy entropy | 0.896 | 0.035 | 0.833 | 0.211 | 739.543 |
| Rényi entropy | 0.885 | 0.037 | 0.861 | 0.263 | 8.919 |

Abbreviation: AUC, Area under the curve;
