# Supplementary figures and images for: Frontal EEG Changes with the Recovery of Carotid Blood Flow in a Cardiac Arrest Swine Model
Source: Sensors (Basel). 2020 May 28;20(11):3052. doi: 10.3390/s20113052 (PMC7313692; doi:10.3390/s20113052)

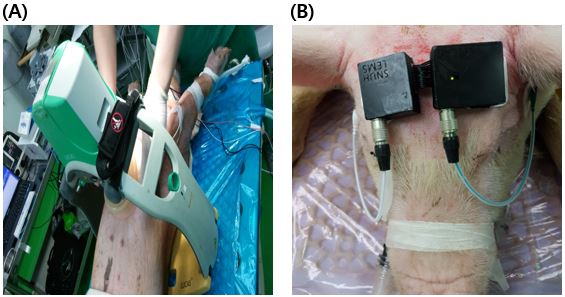

Supplement: Supplementary file 1 [file sensors-20-03052-s001.zip › Sensors2nd_Fig1_300dpi_May25.JPG]

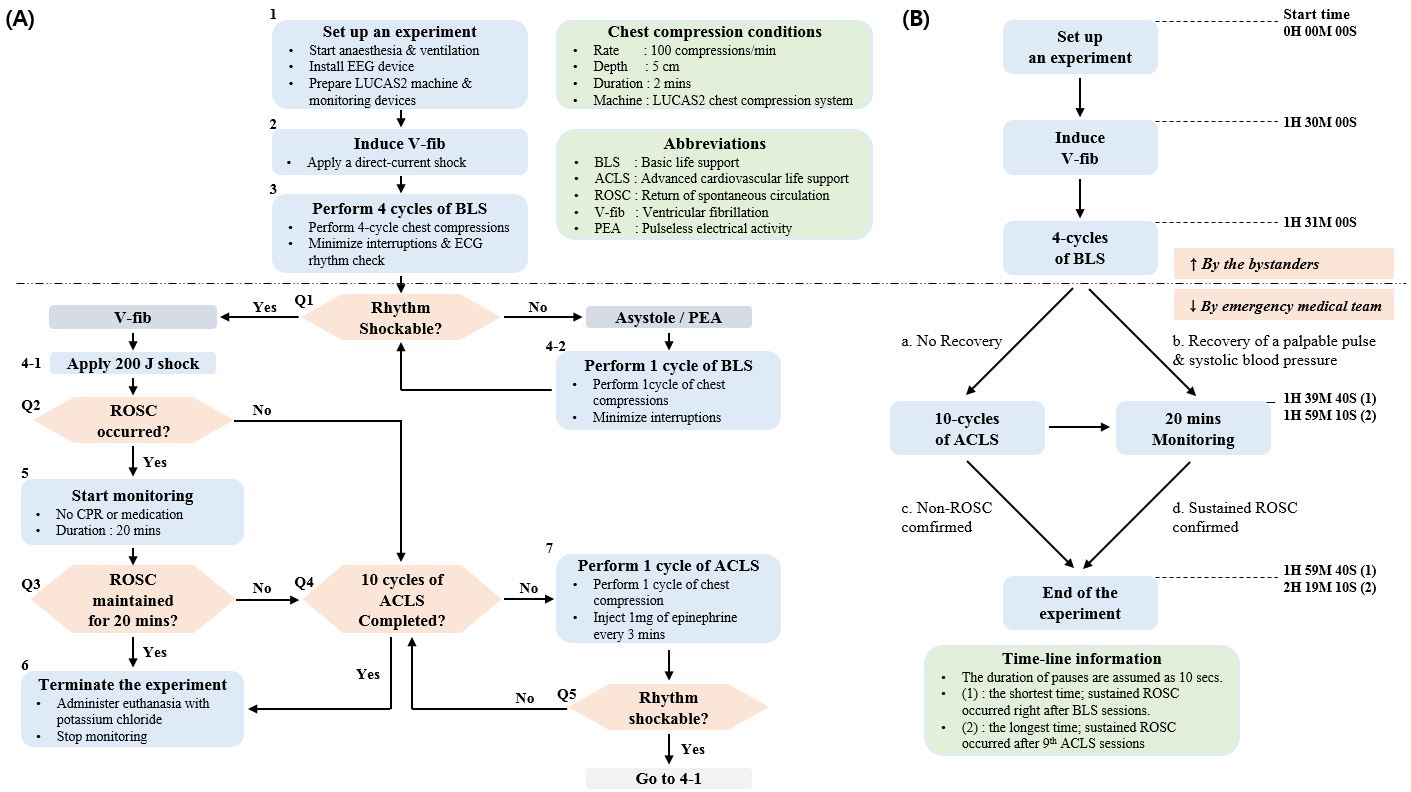

Supplement: Supplementary file 1 [file sensors-20-03052-s001.zip › Sensors2nd_Fig2_300dpi_May25.JPG]

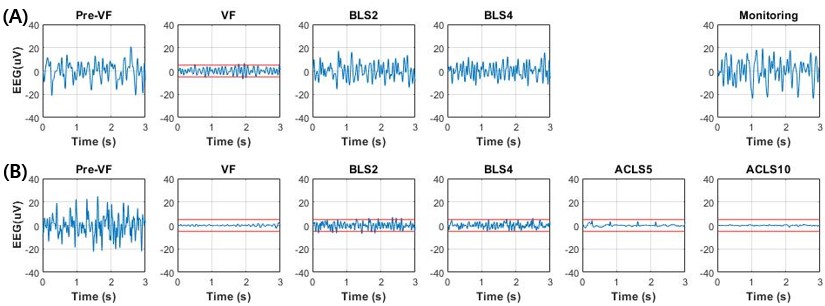

Supplement: Supplementary file 1 [file sensors-20-03052-s001.zip › Sensors2nd_Fig3_300dpi_May25.jpg]

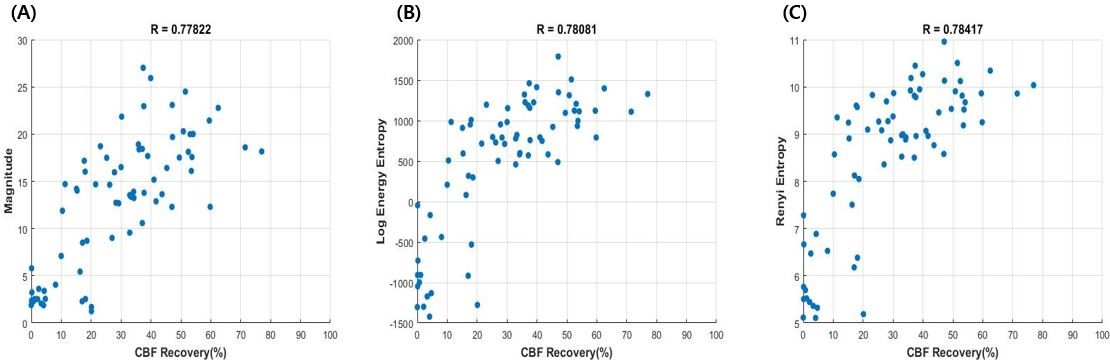

Supplement: Supplementary file 1 [file sensors-20-03052-s001.zip › Sensors2nd_Fig4_300dpi_May25.jpg]

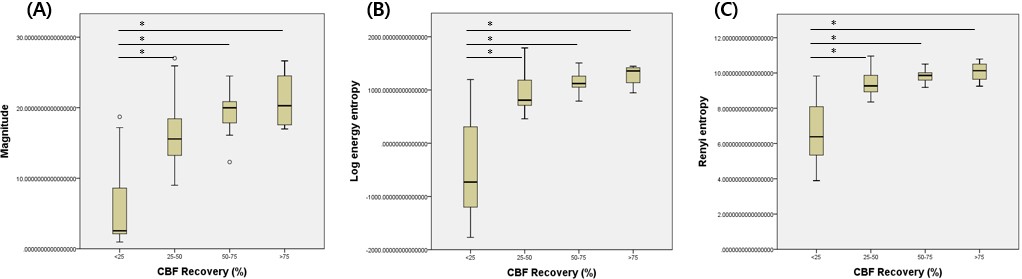

Supplement: Supplementary file 1 [file sensors-20-03052-s001.zip › Sensors2nd_Fig5_300dpi_May25.jpg]

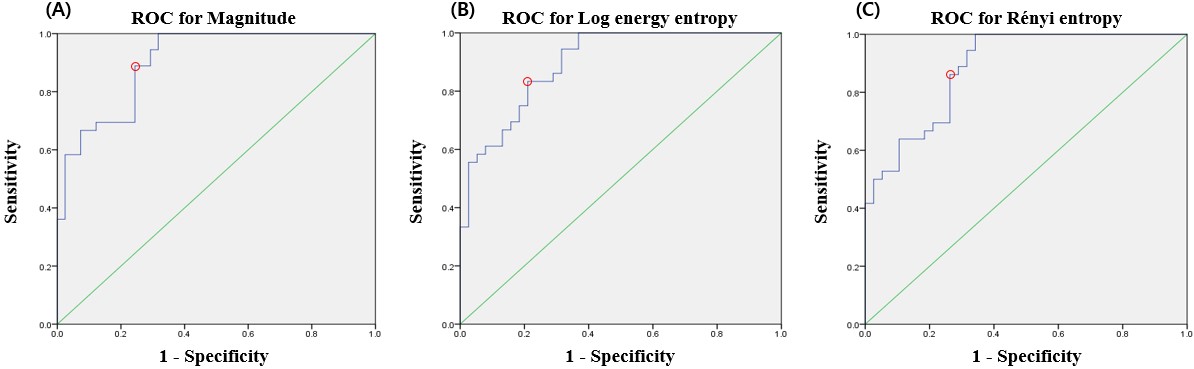

Supplement: Supplementary file 1 [file sensors-20-03052-s001.zip › Sensors2nd_Fig6_300dpi_May25.jpg]
